# Mangiferin inhibits cGAS-STING pathway-related inflammation via Nrf2 activation to protect against sepsis-induced heart injury

**DOI:** 10.1186/s13020-026-01329-9

**Published:** 2026-01-23

**Authors:** Junna Song, Meng Wang, Qian Li, Wanting Zhao, Xiangming Chen, Chunhua Li, Qingmei Guo

**Affiliations:** 1 School of Pharmacy, Shandong University of Traditional Chinese Medicine, Jinan, Shandong China; 2 College of Pharmacy, Hebei University of Chinese Medicine, Shijiazhuang, Hebei China; 3 College of Life Science, Hebei Normal University, Shijiazhuang, Hebei China; 4 Shandong Key Laboratory of Digital Traditional Chinese Medicine, School of Pharmacy, Shandong University of Traditional Chinese Medicine, Jinan, Shandong China

**Keywords:** Heart sepsis, Mangiferin, MtDNA, CGAS-STING pathway, Nrf2

## Abstract

**Background:**

Septic cardiomyopathy is characterized by oxidative stress and inflammation, and accounts for its associated high mortality. Mangiferin is a naturally occurring xanthonoid found abundantly in *Anemarrhena* *asphodeloides* Bunge, a traditional Chinese herb widely used for treatment of cardiovascular diseases. This study was designed to investigate the cardioprotective role of mangiferin against sepsis-induced heart injury with a focus on mitochondrial DNA (mtDNA) release and cGAS-STING pathway-related inflammation.

**Methods:**

The septic cardiomyopathy model in mice was established by intraperitoneal injection of LPS (10 mg/kg). Cardiac Nrf2 in septic mice was knocked down with AAV9-CTNT-Nrf2 shRNA to confirm the activity of mangiferin. Cardiomyocytes were cultured with LPS for further in vitro studies.

**Results:**

Oral administration of mangiferin enhanced the survival of mice against endotoxin-induced insult. When LPS challenge impaired cardiac structural integrity, mangiferin reduced macrophage recruitment in the heart and inhibited the gene expression of pro-inflammatory cytokines. In the septic heart, mangiferin increased Nrf2 protein expression, thereby protecting the heart from oxidative damage. Mechanistically, mangiferin increased Nrf2 protein abundance by promoting Keap-1 degradation, which in turn prevented Nrf2 from undergoing proteasomal degradation. Unlike nuclear DNA (nDNA), mitochondrial DNA (mtDNA) acts as a ligand to induce toll-like receptor (TLR) activation once released into the cytoplasm. By protecting mitochondrial membrane integrity, mangiferin combated oxidative stress to prevent mitochondrial fragmentation and prevented the opening of mitochondrial permeability transition pore (mPTP) and the collapse of mitochondrial membrane potential in a manner this is dependent on Nrf2 availability. These effects were, however, blocked in the presence of a special Nrf2 inhibitor, ML385. Similar to TLR4, TLR9 is a member of the damage-associated molecular patterns (DAMPs). It can induce immune response through STING/IRF3 signaling. In septic mouse heart, mangiferin inhibited cGAS activity, deactivated STING/IRF3 signaling via dephosphorylation and resultantly suppressed interferon response due to limited mtDNA leakage. In cultured cardiomyocytes, mangiferin blocked STING/IRF3 signaling cascades in a Nrf2-dependent manner. Cardiac knockdown of Nrf2 with AAV9-CTNT-Nrf2 shRNA in septic mice demonstrated that Nrf2 deficiency diminished the inhibitory effects of mangiferin on cGAS-STING pathway-related inflammation.

**Conclusion:**

Through Nrf2 activation, mangiferin ameliorates mitochondrial dysfunction to block mtDNA release and subsequent cGAS-STING pathway-related inflammation, resultantly protecting the heart against septic insult. These events suggest the potential in the treatment of heart injury from the perspective of mitochondrial protection.

## Background

Sepsis is induced by dysregulated immune responses when one or more organs damage(s), with septic cardiomyopathy considered as the leading cause of the high mortality associated with this condition [1]. Because more pathological factors are involved in septic heart injury, therapeutics simply targeting specific inflammatory cytokines have failed in clinical trials [2]. Therefore, continuous research in the field should be aimed at finding novel alternatives that target both the immune and non-immune components of sepsis pathogenesis. Recently, emerging evidence revealed the implication of mitochondrial disorders in inflammatory responses to septic heart injury.

Cardiomyocytes are characterized by a high density of mitochondria tightly packed between the sarcomeres. Mitochondria are intracellular organelles with a double membrane, and this structure is essential to ensure membrane integrity and oxidative phosphorylation (OXPHOS). The principal mechanisms involved in mitochondrial dysfunctions in septic cardiomyopathy include overproduction of reactive oxygen species (ROS) and calcium overload [3]. Oxidative stress inhibits mitochondrial biogenesis and impairs respiratory chain proteins, leading to reduced OXPHOS and ATP production [3, 4]. When either inner or outer membrane permeability barriers are disrupted, mitochondria shapes change and this is associated with mitochondrial dysfunction and the release of intracellular contents into the cytoplasm [4].

Different from other intracellular organelles, mitochondria have an independent genome that encodes 37 genes, including 13 genes that encode respiratory chain proteins [5]. When oxidative stress and inflammation impair mitochondrial integrity, the damaged mitochondrial DNAs (mtDNA) are released into the cytoplasm due to the increased membrane permeability.

Mitochondrial DNA itself is a circular molecule of double stranded DNA. When released into the cytoplasm, mtDNA is sensed as “foreign”, because it is seen differently to “self” DNA (nuclear DNA) in cells [6]. Despite the presence of DNA methyltransferases in mitochondria, mtDNA is hypomethylated compared to nuclear DNA [7]. When released from oxidative stressed mitochondria, cyclic GAMP-AMP synthase (cGAS) binds to mtDNA and mediates the conversion of ATP and GDP to cyclic GMP-ATP (cGAMP). As a second messenger, cGAMP binds to the endoplasmic reticulum-resident protein residues of interferon genes (STING) with the recruitment of TANK-binding kinase 1 (TBK1). This process then phosphorylates the transcription factor interferon regulatory factor 3 (IRF3) to elicit the transcription of hundreds of interferon genes, resulting in Type-I interferon response [6]. In mice, lipopolysaccharide (LPS) challenge was found to activate the STING-IRF3 pathway, leading to cardiac dysfunction, inflammation and apoptosis [7]. Consistently, extracellular mtDNA was shown to activate NF-κB signaling in cardiomyocytes [8].

Mangiferin is a naturally occurring xanthonoid derivative which is usually isolated from the mango tree and *Iris* *unguicularis*. The traditional Chinese herb *Anemarrhena* *asphodeloides* Bunge, widely used for the treatment infectious diseases, is a rich source of mangiferin [9]. Mangiferin has been reported to suppress oxidative stress and inflammation via Nfr2 activation [10–12], while other studies have revealed its cardioprotective effect [13, 14]. Because oxidative stress is associated with inflammation, this study sought to investigate the cardioprotective function of mangiferin in septic mice from the viewpoint of oxidative stress-associated inflammation. The results showed that via Nrf2 activation, mangiferin ameliorated mitochondrial dysfunction to prevent mtDNA release, and protected the heart against septic insult.

## Methods

### Animals and treatments

Male C57BL/6 J mice (6–8 weeks old, 20 ± 2 g) were purchased from Beijing Huafukang Biotechnology Co., Ltd. All animals were housed in standard animal cages and had free access to food and water. The housing conditions included light/dark cycling for 12 h, 23 ± 2 ℃ and 45–55% humidity. All animal experiments were approved by the Ethics Review Committee of the Laboratory Animal Center of Hebei University of Traditional Chinese Medicine (Approval number: DWLL202203037).

Drug administration was by oral delivery (gavage). For the determination of mice survival, mangiferin was orally administered to mice (50 mg/kg), 1 h before LPS challenge at 30 mg/kg by intraperitoneal injection as well as 12 and 24 h after LPS stimulation. After challenge, mangiferin was given again at 12 and 24 h in a similar way. The mortality of mice was recorded within 72 h. NAC, an antioxidant agent, was used as the positive control in the experiments (100 mg/kg). The selected dose of mangiferin was based on our previous work, which showed that oral administration of mangiferin at 50 mg/kg effectively protected the heart against aortic construction insult. [15].

Because septic cardiomyopathy is considered to be the leading cause of high mortality in sepsis, we prepared inflammatory heart injury model in mice. Mangiferin (20, 50 mg/kg) was orally administered 1 h before LPS challenge (10 mg/kg intraperitoneal injection) and mice were euthanized for organ (heart) and blood (plasma) collection.

For in vitro experiments, cells were treated with LPS (1 μg/mL) for 24 h and then examined for intracellular inflammatory cytokines and antioxidative enzymes. The intracellular inflammatory cytokines and antioxidative enzymes were assayed in a concentration- and time-dependent manner.

For Nrf2 knockdown in the heart, mice were randomly chosen to receive a single-bolus tail vein injection of either AAV9-CTNT-Ctrl or AAV9-CTNT-Nrf2 shRNA (Hanbio Biotechnology, Shanghai, China) at 1*10^11^ vg (viral genomes) per mice for 4 weeks. After examining the efficiency of knockdown, the model mice were subjected to LPS injection (10 mg/kg, intraperitoneally), and their hearts harvested after 24 h. Mice were orally administered mangiferin (50 mg/kg) 2 h before LPS challenge, followed by another administration 12 h after LPS treatment (10 mg/kg). After 24 h, the hearts and plasma of the mice were gotten. For Nrf2 knockdown in the heart, we utilized AAV9 vector carrier with CTNT promoter (AAV9-CTNT-shNrf2) [16, 17]. The treated mice were divided into four groups: transfection blank control (blank), LPS stimulation, LPS via mangiferin, LPS via mangiferin via AAV9-CTNT-Nrf2-shRNA*.* In cell culturing Nrf2 knockdown was treated in the same way.

### Cell culture and treatments

H9c2 cardiomyocytes were purchased from the Stem Cell Bank, Chinese Academy of Sciences. Cells were cultured in DMEM (containing 10% FBS) in a cell incubator (37 ℃, 5% CO_2_). For LPS challenge, cells were cultured with LPS (1 μg/mL) for 24 h.

### Cardiac ultrasound

Mice were anesthetized via inhalation of isoflurane using a specialized rodent anesthesia induction system. Following the induction of anesthesia, the mice were positioned in a supine posture and securely immobilized.

### Transmission electron microscopy

To observe the morphology of mitochondria in myocardial cells, isolated heart tissues were rapidly fixed with electron microscope fixation solution at 4 °C for 2–4 h. After fixation, the tissues were placed in ethanol and acetone for gradient dehydration. The mitochondrial structure was viewed using the transmission electron microscope (HITACHI, Japan) for image acquisition and analysis.

### Histopathology and immunohistochemistry of heart tissues

Fresh heart tissues were fixed in 4% paraformaldehyde (Sigma-Aldrich, catalog no. P6148), paraffin-embedded, and sectioned into 4-μm slices for staining with hematoxylin and eosin (HE, Servicebio, catalog no. G1003). The image was observed using a SZ61 zoom type microscope (Olympus, Japan) and the VHX-6000 Keyens Digital Imaging system (Keyence, Japan).

For macrophage recruitment determination in the injured heart, we used immunohistochemical staining probe. The fixed sections were stained with rabbit anti-mouse F4/80, rabbit anti-mouse TLR9 primary antibody and goat anti-rabbit fluorescent secondary antibodies, respectively. ROS production in the tissue was labeled with DCFH-DA probe. After sealing, images were observed and acquired using an LSM800 confocal laser microscope (Zeiss, Germany).

### Assay of biochemical parameters

The contents of 8-OHdG, Bax and Cytc, as well as PARP-1 and cGAS in heart tissues or in cultured H9c2 cells were detected using commercial ELISA kits (Ruifan, China). Circulating lactate dehydrogenase activity (LDH) and inflammatory cytokines were assayed with commercial Kits (Nanjing Jiancheng, China).

### Immunofluorescence staining in cultured cells

H9c2 cells were stimulated with LPS (1 μg/mL) for 24 h. The intracellular ROS and mitochondrial membrane potential (Δψm) were determined by scanning with a microplate reader after loading with DCFH-DA (Beyotime, S0033S) and potentiometric dye 500 nM TMRE (Beyotime, C2001S), respectively. The mitochondrial permeability transition Pore (mPTP) opening was detected under a Zeiss 880 confocal microscope using a mPTP Fluorescence Assay Kit (Beyotime, China). The fluorescence intensity was analyzed using the Image software.

### Western blotting analysis

Total proteins were extracted by lysing mouse myocardial tissue and H9c2 cells in RIPA buffer (Solarbio, China). The protein concentration was determined using a BCA assay kit. Protein was subjected to sodium dodecyl sulfate–polyacrylamide gel electrophoresis (SDS-PAGE) and then transferred to a PVDF membrane. PVDF membranes were immersed in 5% skim milk powder and incubated for 2 h. The membrane was then incubated with the individual primary antibodies at 4 ℃ overnight. The primary antibodies were anti-Nrf2 (1:800, Abways, CY5136); anti-TLR9 (1:1000, Affinity, DF2970); anti-p-NF-κB (Ser536) (1:1000, Abways, CY6372); anti-NF-κB (1:1000, Affinity, AF5006); anti-p-IRF3 (Ser396) (1:1000, Affinity, AF2436); anti-IRF3 (1:1000, Affinity, DF6895); anti-p-STING (Ser366) (1:1000, Affinity, AF7416); anti-STING, (1:1000, Affinity, DF12096); anti-DRP1, (1:1000, Abways, AY2001); anti-GAPDH, (1:5000, Abways, AB0036); anti-α-Tubulin, (1:5000, proteintech, 11224-1-AP). The PVDF membrane was washed with 1 × TBST solution and incubated with secondary antibody horseradish peroxidase (HRP)-conjugated goat antirabbit IgG (1:10000, Abways, F300405) for 1 h. Protein bands were visualized using an ECL (Shenger Biot, China) luminescent solution and a multifunctional imaging system (Vilber, France). Subsequently, ImageJ software was used to analyze the gray values of the protein bands.

### Reverse transcription-quantitative PCR (RT-qPCR)

Total RNA was isolated from mouse myocardial tissue and H9c2 cells according to the instructions of the total RNA extraction kit (Promega, USA). The concentration and purity of RNA were determined by mixing 2 µg of template RNA, the reagent in the reverse transcription kit (Thermo Fisher Scientific, USA), and ribozyme-free water. qRT-PCR was performed using a ChamQ Universal SYBR qPCR Master Mix kit (Vazyme, China). Using GAPDH as an internal control, the 2^−^^ΔΔCt^ formula was used to calculate the relative expression of target genes. The primer sequences are listed in Table 1.

**Table 1:** Primer sequences for qRT-PCR

Primer	Species	Forward primer (5ʹ → 3ʹ)	Reverse primer (5ʹ → 3ʹ)
GAPDH	Mouse	GGTTGTCTCCTGCGACTTCA	TGGTCCAGGGTTTCTTACTCC
HO-1	Mouse	TCCTTGTACCATATCTACACGG	GAGACGCTTTACATAGTGCTGT
Nqo1	Mouse	GAAGACATCATTCAACTACGCC	GAGATGACTCGGAAGGATACTG
mt-ND1	Mouse	CTAATCGCCATAGCCTTCCTAA	GTTGTTAAAGGGCGTATTGGTT
mt-Cytb	Mouse	CCACTCATTCATTGACCTACCT	GCTCCGTTTGCGTGTATATATC
TNF-α	Mouse	AGGGGATTATGGCTCAGGGT	CCACAGTCCAGGTCACTGTC
IL-6	Mouse	CTCCCAACAGACCTG TCTATAC	CCATTGCACAACTCTTTTCTCA
IL-1β	Mouse	CACTACAGGCTCCGAGATGAACAAC	TGTCGTTGCTTGGTTCTCCTTGTAC
CXCL 10	Mouse	CCCAAGTGCTGCCGTCATT	CCCAAGTGCTGCCGTCATT
Ifnb1	Mouse	CACAGCCCTCTCCATCAACTATAAG	TGGATGGCAAAGGCAGTGTAA
GAPDH	Rat	GACATGCCGCCTGGAGAAAC	AGCCCAGGATGCCCTTTAGT
HO-1	Rat	CAGGTGTCCAGGGAAGGCTTTAAG	TGGGTTCTGCTTGTTTCGCTCTATC
Nqo1	Rat	CCATTCCAGCCGACAACCAGATC	CCATTCCAGCCGACAACCAGATC
Ifnb1	Rat	AGAAGAGTTACACTGCCTTTGCC	GTCACCCAAGTCAATCTTTCCTC
CXCL 10	Rat	ATTCCTGCAAGTCTATCCTGTCC	ACCTTCTTTGGCTCACCGCTT

### Mitochondrial permeability transition pore (mPTP)

After treatment, the cells were washed twice with PBS, and a certain volume of fluorescence quenching working liquid (Beyotime, China) was added. The cells were incubated at 37 °C for 40 min in the dark. After incubation, the culture medium was replaced with new preheated culture medium and incubated at 37 ℃ for 30 min in the dark. After adding detection buffer, the green fluorescence intensity was observed and photographed under a fluorescence microscope at 400 × magnification. The fluorescence intensity was analyzed using the ImageJ software.

### Mitochondrial membrane potential (Δψm) and mitochondrial number

To determine the mitochondrial membrane potential (Δψm), cells were incubated with TMRM (Invitrogen™, USA) at 37 ℃ for 10 min in the dark. To quantify mitochondrial numbers, cells were cultured with 0.1 μM nonyl acridine orange (Invitrogen™, USA) at 37 ℃ for 30 min in the dark. Nuclei were stained with a DAPI dye solution. According to the manufacturer’s instructions, the fluorescence intensity of the cells was observed using an LSM800 confocal laser microscope (Zeiss, Germany).

### Mitochondrial DNA copy number in the cytoplasm

The cytoplasm of tissues and cells was extracted using a cell grading kit. DNA was extracted from the cytoplasm using a DNA extraction kit. Representative mtDNA-encoded mt-ND1 and mt-Cytb levels were selected to reflect mtDNA levels, and cytoplasmic DNA copy numbers were detected using the ChamQ Universal SYBR qPCR Master Mix.

### Statistical analysis

Prism Graphpad 8.1 software was used for statistical analysis of the data. Data are expressed as mean ± SEM. One-way analysis of variance was used for multi-group comparisons, and Tukey’s test was used for pairwise comparisons between the groups. P < 0.05 was considered statistically significant.

## Results

### Mangiferin protects mouse heart against endotoxin insult

**Fig. 1:**
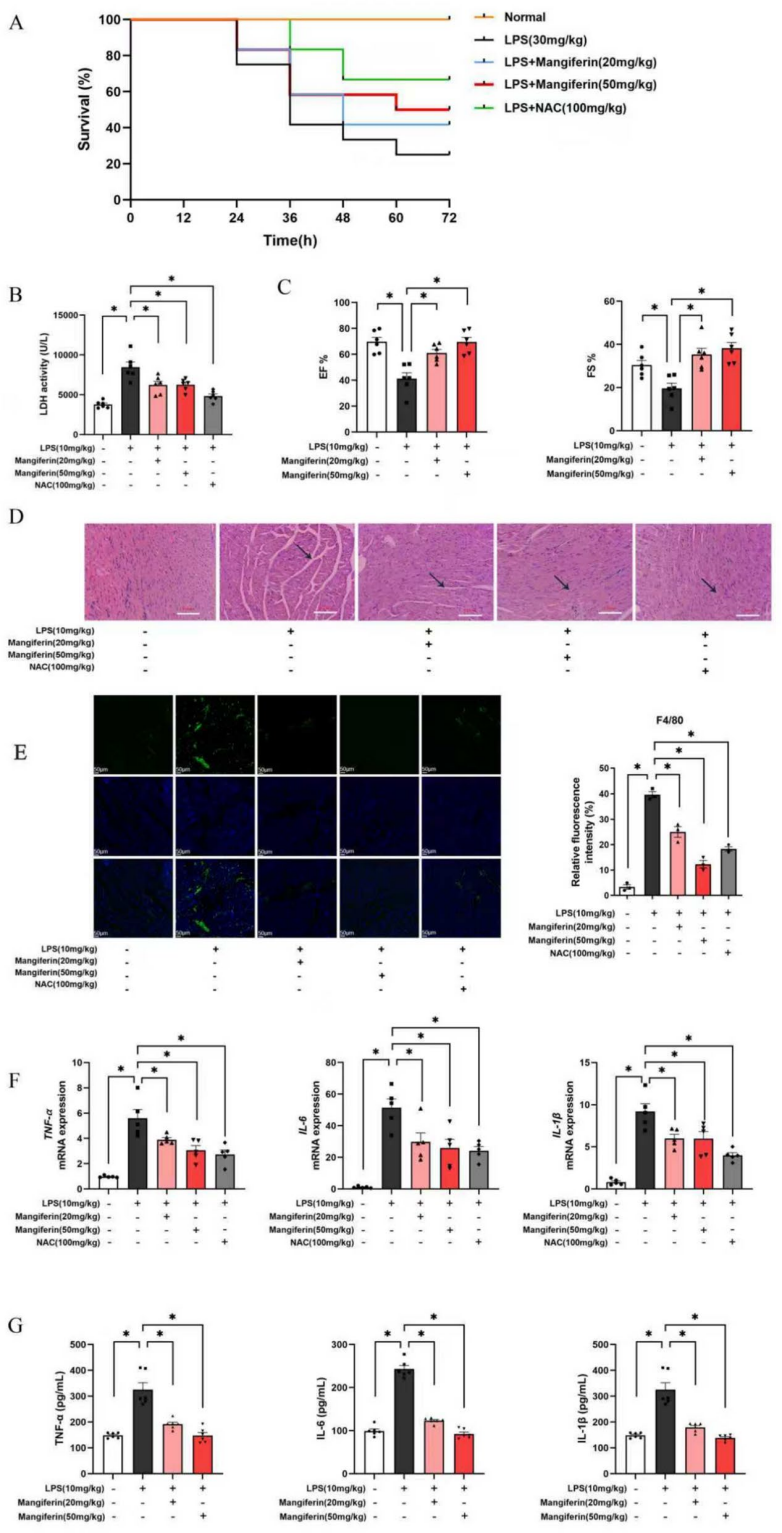
Mangiferin protects the heart against LPS challenge. **A** Mice survival (n = 12). **B** LDH release in the blood (n = 6). **C** cardiac ultrasound in mice **D** HE staining of the heart. **E** F4/80 staining (one of three different experiments). **F** Gene expressions of *TNF-α*, *IL-6* and *IL-1β* in the heart (n = 5). **G** Released *TNF-α*, *IL-6* and *IL-1β* in the mice plasma (n = 5). Data are presented as mean ± SEM. *p < 0.05 (compared with indicated treatments)

We first examined the influence of mangiferin on mice survival. Mice were challenged with LPS (30 mg/kg, intraperitoneally), and mangiferin was orally administered 1 h before LPS challenge, followed by continuous administration at 24 and 48 h (20 mg/kg and 50 mg/kg) after challenge. Mangiferin (50 mg/kg) enhanced mice survival by reducing 72 h mortality from 75 to 50% (Fig. 1A). We then observed the effects of mangiferin on septic heart at 24 h after LPS injection (10 mg/kg), and found that mangiferin effectively reduced elevated levels of LDH in the blood (Fig. 1B). We examined the effects of mangiferin on heart function with cardiac ultrasound, and these results showed that mangiferin administration restored Ejection Fraction (EF) and Fractional Shortening (SF) in a dose-dependent manner, thereby improving heart function (Fig. 1D). F4/80 staining showed that mangiferin reduced macrophage infiltration in the heart (Fig. 1E). Mangiferin attenuated myocardial fiber injury (Fig. 1C). F4/80 staining showed that mangiferin reduced macrophage infiltration in the heart (Fig. 1D). Meanwhile, mangiferin effectively inhibited gene expression of *TNF-α, IL-6* and *IL-1β* in the heart (Fig. 1F). Consistently, blood inflammatory cytokines were also down regulated, which is indicative of suppressed systemic inflammation (Fig. 1G). N-Acetlyl-cysteinN-Acetlyl-cystein (NAC) is an antioxidant agent used here as a positive control for suppression of oxidative response.

### Mangiferin combats oxidative stress in septic heart

**Fig. 2:**
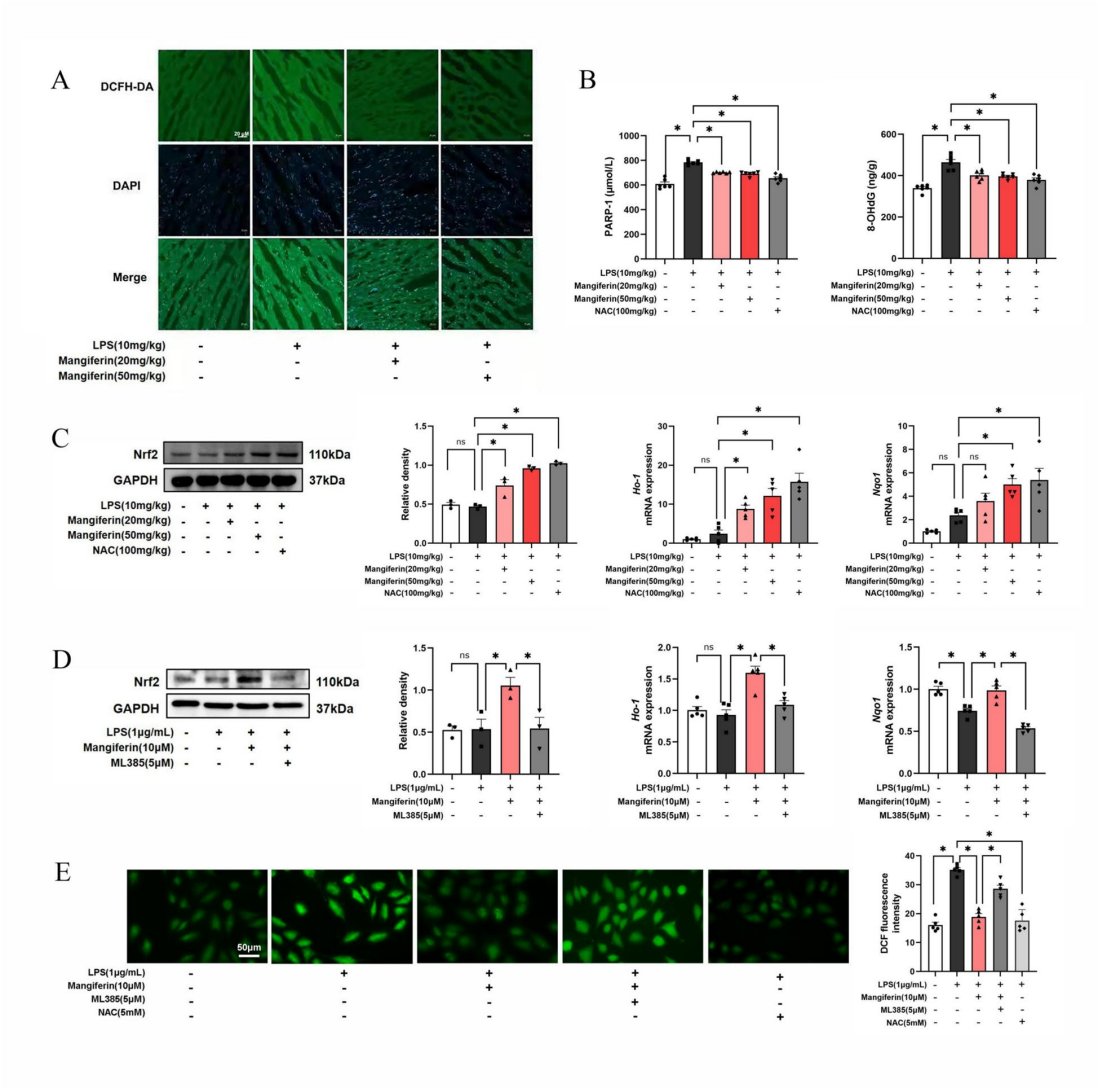
Mangiferin combats cardiac oxidative stress in mouse heart **A** DCFH-DA staining in LPS-challenged heart tissues (one of three different experiments). **B** PARP-1 and 8-OHdG contents in mouse heart (n = 6). **C** Upregulated Nrf2 protein expression (n = 3) and *HO-1*, *Nqo1* mRNA expression in mouse heart (n = 5). **D** Nrf2 protein (n = 3) and *HO-1*, *Nqo1* mRNA expression in H9c2 cells (n = 5). **E** ROS production in H9c2 cells (n = 5). Data are presented as mean ± SEM. *p < 0.05 (compared with indicated treatments)

LPS stimulation elicited oxidative stress, but mangiferin administration suppressed ROS production and reduced PARP-1 and 8-OHdG accumulation in the mouse heart (Fig. 2A, B), an indication of its ability to limit DNA oxidative damage. Mangiferin increased Nrf2 protein abundance accompanied by gene induction of *HO-1* and *Nqo1*, which are Nrf2-related genes responsible for combating oxidative stress (Fig. 2C). In cultured H9c2 cells, mangiferin also increased Nrf2 protein expression and gene induction of *HO-1* and *Nqo1* with reduced ROS production. These actions were, however, blocked by the Nrf2 inhibitor, ML385 (Fig. 2D, E). These results demonstrate that mangiferin combated oxidative stress based on Nrf2 activation.

### Mangiferin increases Nrf2 protein stability via Keap-1 degradation

**Fig. 3:**
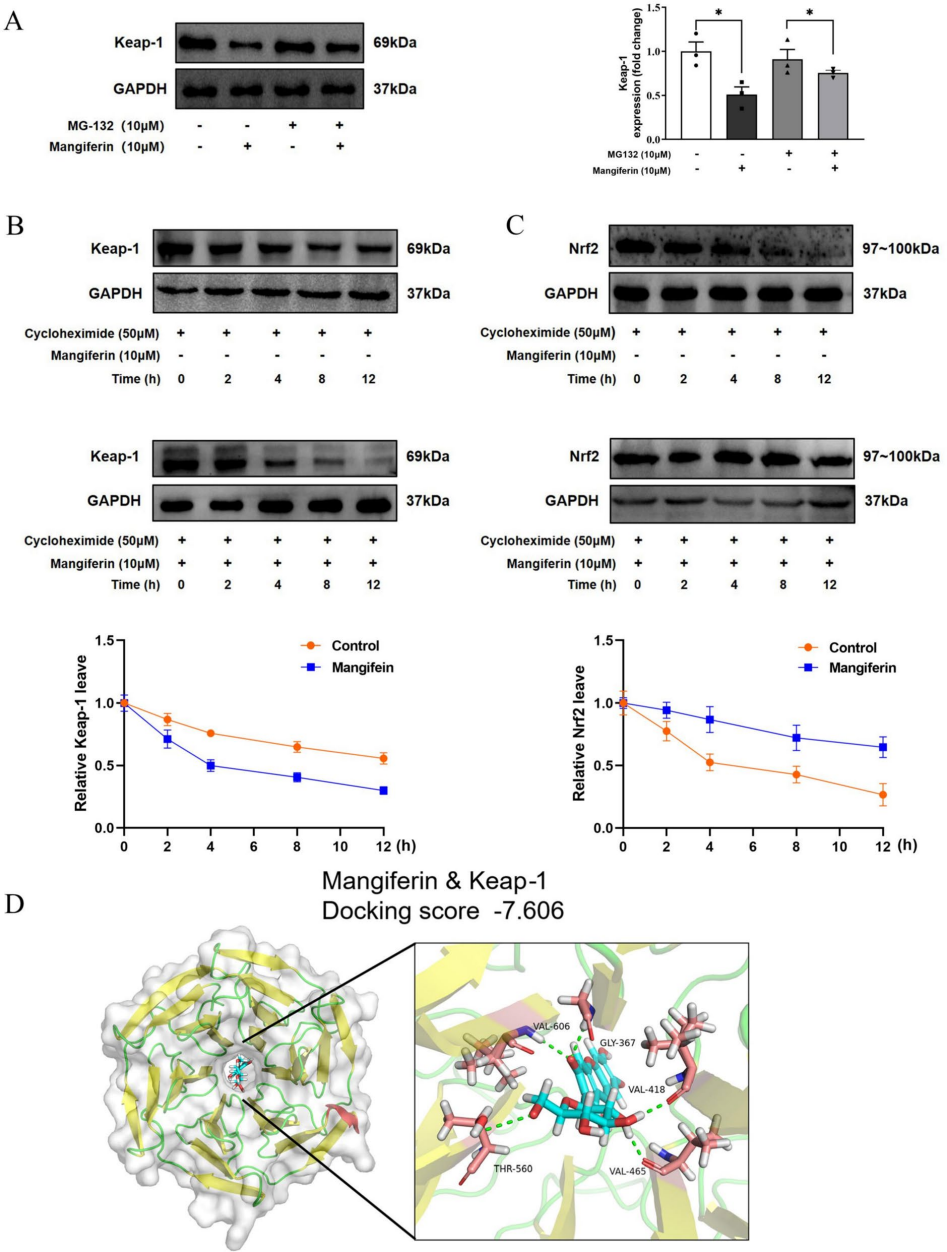
Mangiferin increases Nrf2 protein stability via Keap-1 degradation. **A** Stability of Keap-1 protein in the presence of proteasome inhibitor MG-132 in resting H9c2 cells cultured for 24 h. **B** Keap-1 protein stability when protein synthesis was inhibited by cycloheximide in H9c2 cells cultured with Magiferin for 2–12 h. **C** Nrf2 protein stability in H9c2 cells cultured with Magiferin for 2–12 h in the presence cycloheximide. **D** Molecular docking of mangiferin and Keap-1. Data are presented as mean ± SEM. *p < 0.05 (compared with indicated treatments)

Under normal conditions, the cytoplasmic protein kelch-like ECH-associated protein 1 (Keap-1) binds to Nrf2 to promote Nrf2 degradation through protein ubiquitination [18]. To know if mangiferin promoted Nrf2 protein accumulation via disturbing Keap-1 stability, we determined Keap-1 protein expression. Mangiferin reduced Keap-1 protein abundance, while this effect was diminished by proteasome inhibitor MG-132 (Fig. 3A), suggesting that mangiferin impaired Keap-1 protein stability through proteasome degradation. For confirmation, we inhibited protein synthesis in cells with cycloheximide and found that Keap-1 protein was expressed in a steady state, but the stability was impaired by mangiferin treatment, as shown by continuous degradation from 2–12 h (Fig. 3B). In contrast, Nrf2 protein was continuously degraded from 2–12 h and mangiferin treatment prevented Nrf2 protein degradation (Fig. 3C). Moreover, we conducted molecular docking to determine the interaction between mangiferin and Keap-1 protein. The results showed that mangiferin interacted with keap-1 through five hydrogen bonds at the following active site residues, GLY367, VAL418, VAL465, THR560 and VAL606 to achieve a binding score of −7.606 kcal mol^−^^1^ (Fig. 3D). These results provide evidence in support of the assertion that mangiferin promotes Keap-1 degradation to improve Nrf2 protein stability.

### Mangiferin protects mitochondrial integrity

**Fig. 4:**
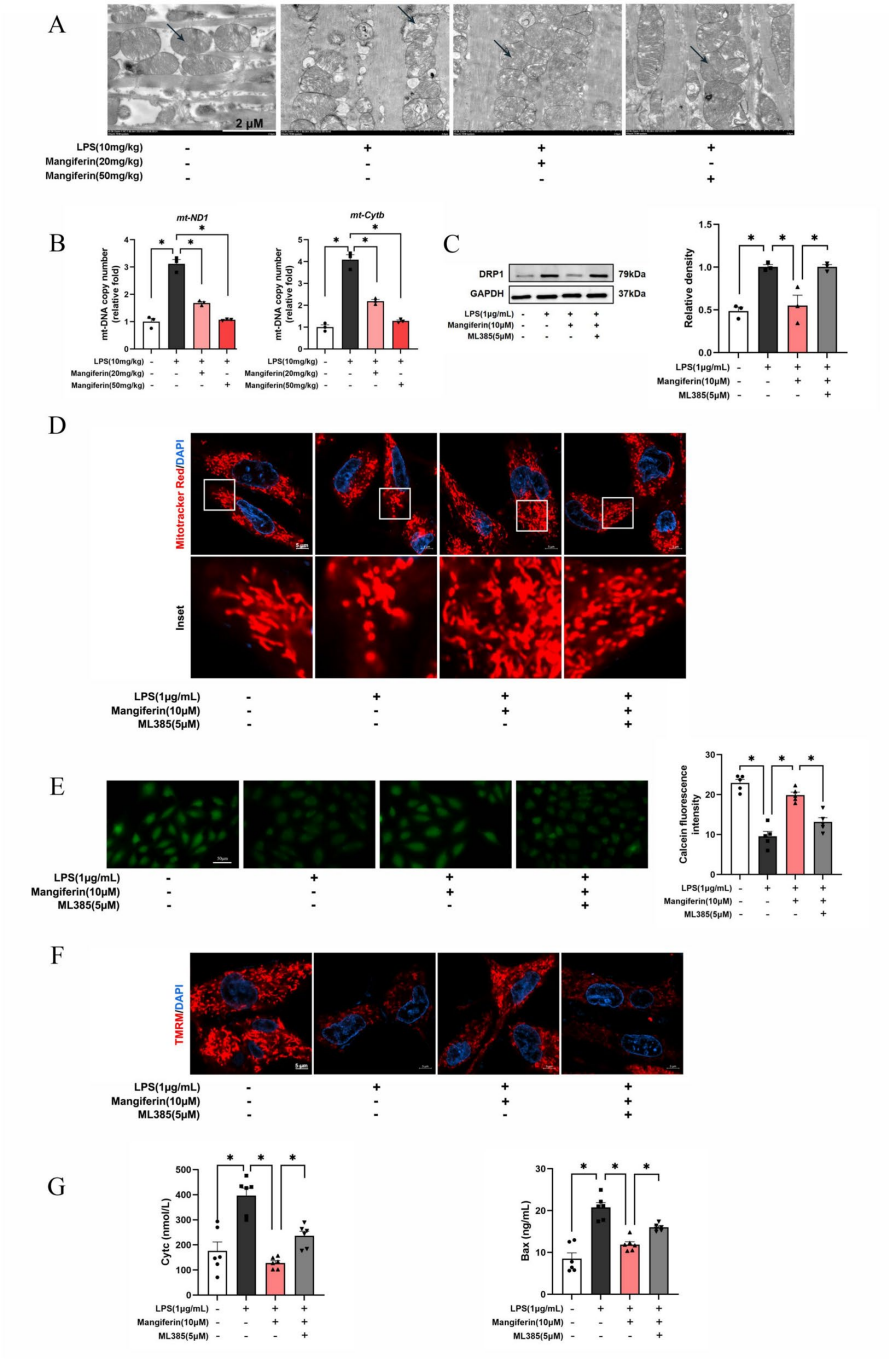
Mangiferin protects mitochondrial integrity. **A** Mitochondrial morphology of mouse heart tissue (one of three different experiments). **B** mtDNA copy number in mouse heart tissue (n = 3). **C** DRP1 protein expression in H9c2 cells (n = 3). **D** Number of mitochondria in H9c2 cells (n = 5). **E** Changes in mitochondrial Permeability Transition Pore (mPTP) opening in H9c2 cells (n = 5). **F** Mitochondrial membrane potential in H9c2 cells (n = 5). **G** Contents of Cytc and Bax in H9c2 cells (n = 6). Data are presented as mean ± SEM. *p < 0.05 (compared with indicated treatments)

A view of the transmission electron microscope images showed that LPS challenge induced notable cytoarchitectural aberrations in mitochondria, indicated by decline of mitochondrial density, formation of internal vesicles and loss of cristae, all of which were normalized in the mangiferin-treated mice (Fig. 4A). As a result of mitochondria protection, mangiferin reduced mtDNA release into the cytoplasm in the heart tissue (Fig. 4B). In response to stress, DRP1 protein is translocated from the cytoplasm to mitochondrial outer membrane mediating mitochondrial fission. Mangiferin inhibited DRP1 protein expression in LPS-stimulated cardiomyocytes (Fig. 4C). Mito tracker red probe showed that LPS stimulation reduced mitochondrial number and evoked mitochondrial fragments, whereas these alterations were reversed by mangiferin in a Nrf2-dependent manner (Fig. 4D). As expected, mangiferin prevented the opening of mitochondrial permeability transition pore (mPTP) and restored the collapse of mitochondrial membrane potential in Nrf2-dependent manner (Fig. 4E, F), thereby protecting mitochondrial integrity. As a result, mangiferin reduced Cytc and Bax release from mitochondria against apoptosis (Fig. 4G).

### Mangiferin suppresses mtDNA-relevant cardiac inflammatory responses

**Fig. 5:**
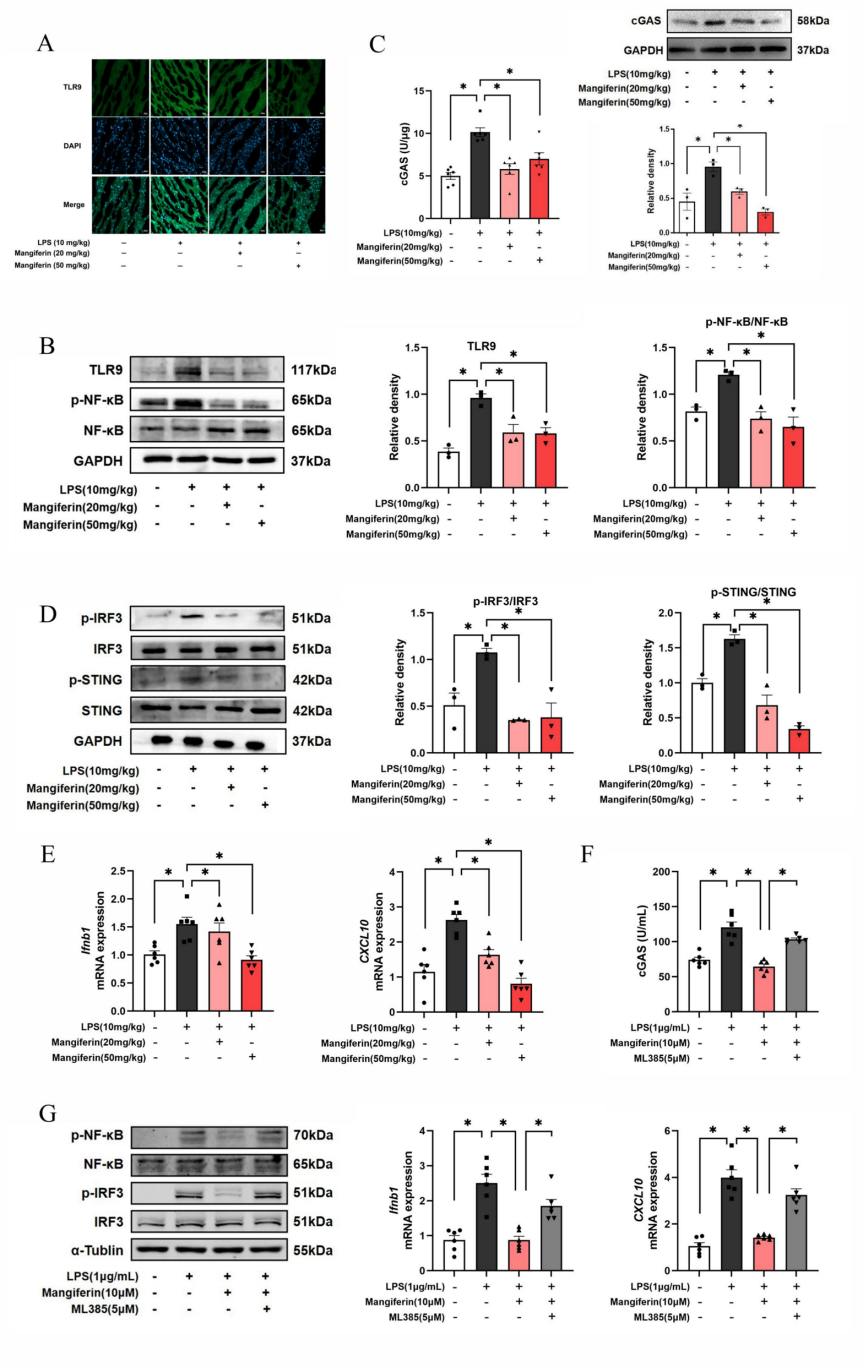
Mangiferin suppresses mtDNA-relevant cardiac inflammatory responses. **A** TLR9 fluorescence staining in mouse heart tissue. **B** TLR9 protein expression and phosphorylation of NF-κB in mouse heart tissue (n = 3). **C** cGAS activity in mouse heart (n = 6). Protein expression of cGAS in mouse heart (n = 3). **D** Phosphorylation of STING and IRF3 in mouse heart (n = 3). **E** *Ifnb1* and *CXCL10* mRNA expression in mouse heart (n = 6). **F** cGAS activity in H9c2 cells (n = 6). **G** Phosphorylation of NF-κB and IRF3 in H9c2 cells (n = 3), *Ifnb1* and *CXCL10* mRNA expression in H9c2 cells (n = 6). Data are presented as mean ± SEM. *p < 0.05 (compared with indicated treatments)

Unlike TLR4, TLR9 is a damage-associated molecular patterns (DAMPs) protein that is involved in innate immune responses [19]. Immunofluorescence staining showed highly expressed TLR9 in the heart but normalized by mangiferin administration (Fig. 5A). In support, western blotting demonstrated that mangiferin reduced TLR9 protein expression. More than activation of TLR4, mtDNA also activates TLR9 to activate NF-kB and impair mitochondria integrity through TLR9/NF-kB signaling. Mangiferin inactivated NF-kB via dephosphorylation in the hearts of mice subjected to LPS insult (Fig. 5B), thus blocking TLR9/NF-kB signaling. Meanwhile, mangiferin reduced cGAS activity with protein expression suppression (Fig. 5C) and blocked STING pathway by inactivating STING and IRF3 via dephosphorylation (Fig. 5D), resultantly inhibiting interferon genes (*Ifnb1* and *CXCL10*) expressions (Fig. 5E). Similarly, in LPS-stimulated cardiomyocytes, mangiferin inhibited cGAS activity and protein expression and inactivated NF-kB and IRF3 via dephosphorylation (Fig. 5F, G), thus suppressing the gene induction of interferon cytokines (Fig. 5H). As the inhibitory effects were diminished by Nrf2 inhibitor ML385, these results suggest that mangiferin inhibited cardiac cGAS-STING pathway-mediated interferon response in a Nrf2-dependent manner.

### Nrf2 deficiency attenuates the inhibitory effects of mangiferin on STING pathway-mediated inflammation in mouse heart

**Fig. 6:**
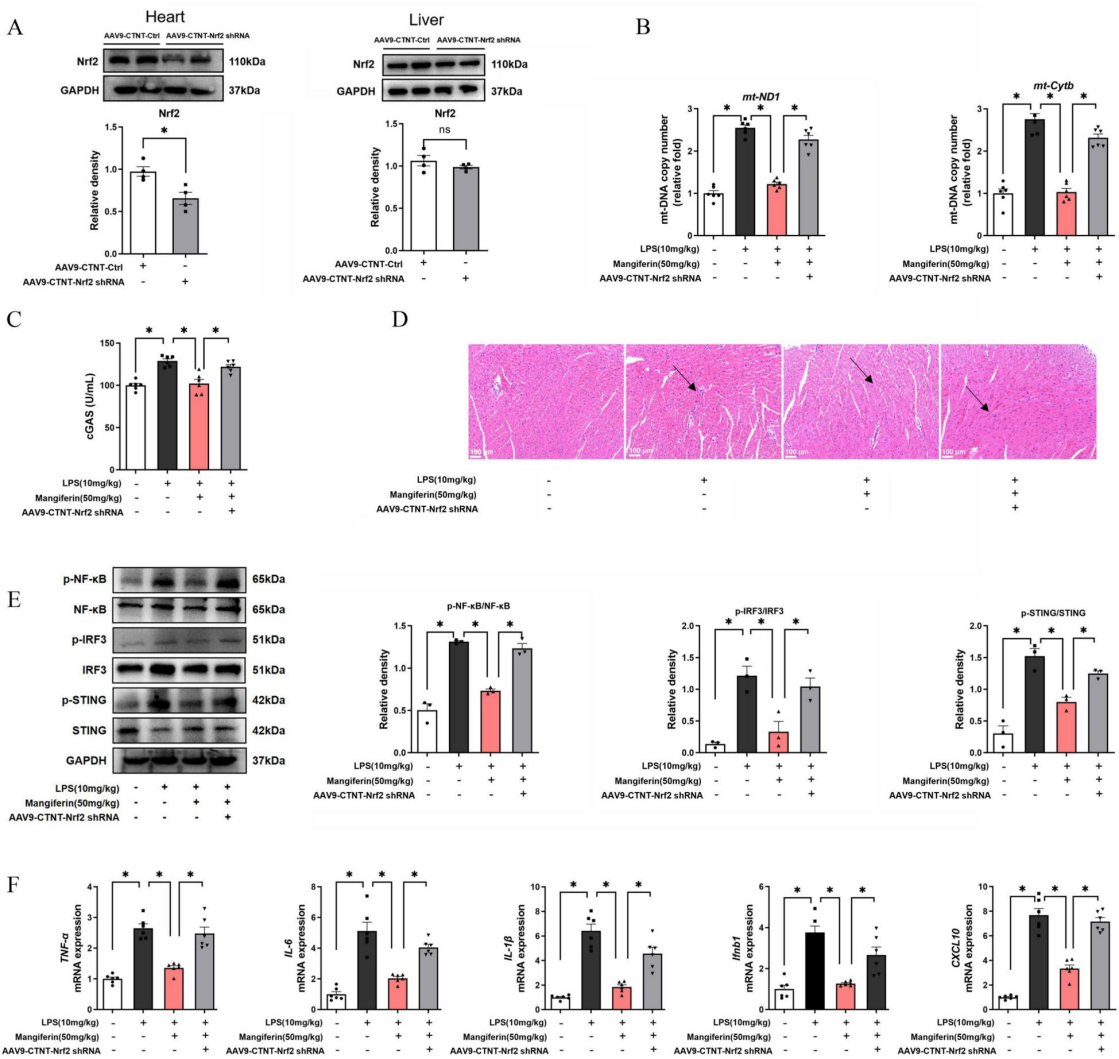
Nrf2 deficiency attenuates the inhibitory effects of mangiferin on STING pathway-related inflammation in mouse heart. **A** Nrf2 protein expression in the heart and liver of Ctrl and shNrf2 mice (n = 3). **B** mtDNA copy number in heart tissue (n = 6). **C** The viability of cGAS in mouse heart (n = 6). **D** HE staining of mouse heart **E** Phosphorylation of NF-κB, IRF3 and STING in mouse heart (n = 3). **F** *TNF-α, IL-1β, IL-6, Ifnb1* and *CXCL10* mRNA expression in mouse heart (n = 6). Data are presented as mean ± SEM (n = 6). *p < 0.05 (compared with indicated treatments)

To address the importance of Nrf2 in the activity of mangiferin, we silenced cardiac Nrf2 in mice by tail vein injection of AAV9-CTNT-Nrf2 shRNA prior to LPS challenge (Fig. 6A). For Nrf2 knockdown in the heart, we utilized AAV9 vector carrier with CTNT promoter (AAV9-CTNT-shNrf2) to ensure specific Nrf2 knockdown in the mouse heart. The results showed that Nrf2 knockdown was actually specific for the heart and other organs such as the liver were not affected. Mangiferin reduced mtDNA contents released in the heart cytoplasm with cGAS inactivation, but the effects were attenuated in Nrf2-deficient mouse heart (Fig. 6B, C). HE staining showed that mangiferin attenuated heart structural injury, and its effects were attenuated after Nrf2 knockdown (Fig. 6D). Mangiferin inactivated STING, IRF3 and NF-κB via dephosphorylation in a manner that was dependent on Nrf2 availability (Fig. 6E). As a result, mangiferin failed to inhibit inflammatory and interferon responses in Nrf2-deficient mice hearts (Fig. 6F). These results provided evidence in support of the fact that Nrf2 induction is required for mangiferin to block cardiac interferon responses.

## Discussion

**Fig. 7:**
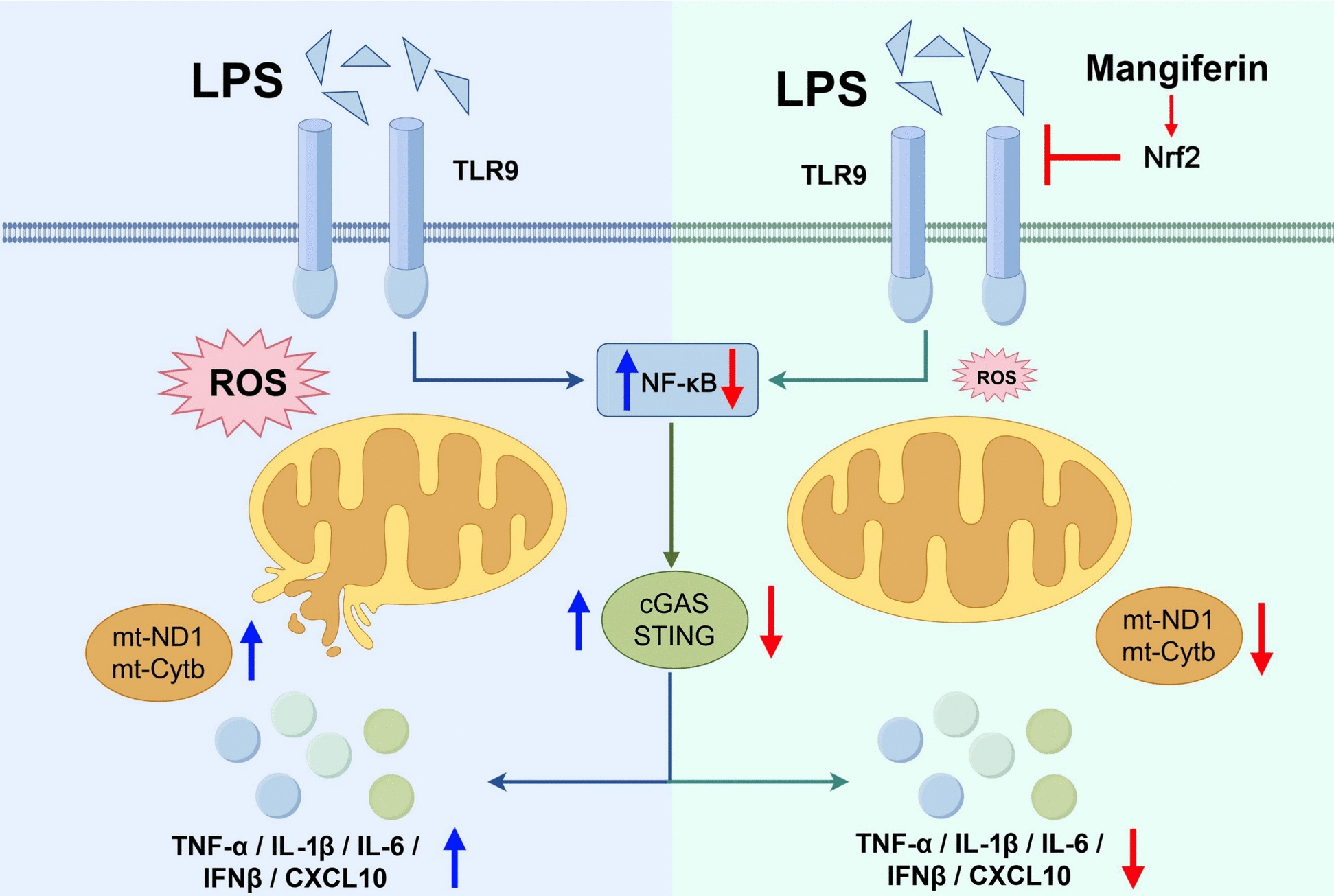
Schematic diagram for mechanism for cGAS/STING signaling

Septic heart injury is tightly associated with oxidative stress and inflammation. Aside from anti-oxidative stress activity, accumulating evidence demonstrates that Nrf2 also possesses the ability to inhibit inflammation [19]. Genetic or pharmacological activation of Nrf2 has been found to suppress acute inflammatory liver injury [20] and neuroinflammation [21]. Related studies have reported that mangiferin inhibits inflammation with Nrf2 upregulation though the underlying mechanism(s) remained unclear [11, 12]. In the present study, we demonstrate that by activating Nrf2, mangiferin protects mitochondria against oxidative stress and resultantly blocks mtDNA-mediated interferon response in septic mouse heart (Fig. 7). These findings provide novel information on the role of mangiferin in the control of oxidative stress.

illustrating the mechanism of action for mangiferin in combating septic heart injury. Upon endotoxin insult, TLR9 activates and impairs mitochondrial integrity and thus promotes mtDNA release. By activating Nrf2, mangiferin combats oxidative damage and protects mitochondrial membrane permeability against mtDNA leakage, thereby blocking cGAS-STING cascades-mediated inflammation.

Septic heart is associated with oxidative stress. It is well known that LPS increases ROS production mainly from NADPH oxidase in activated macrophages [22]. However, it was recently revealed that LPS promotes ROS production from mitochondria due to reverse electron transport [23]. In response to septic insult, mitochondrial ATP synthase is inhibited with succinate accumulation and upregulation of succinate dehydrogenase activity to drive electron transport in reverse and promote ROS production from mitochondrial Complex I and III [24]. Mangiferin activates Nrf2, which transcriptionally upregulates genes encoding antioxidant and detoxifying enzymes such as SOD2 and catalase in the mitochondria to scavenge mitochondrial ROS. Mangiferin combats oxidative and inflammatory damage and contributes to protecting cardiac systolic function.

Mitochondrial disorders are involved in septic cardiomyopathy, characterized by ultrastructural abnormalities, such as swelling and disruption of cristae. Mitochondria are organelles that undergo fusion and fission in a dynamic manner. In response to stress, DRP1 protein is translocated to mitochondrial outer member and mediates mitochondrial fission. In this study, mangiferin protected mitochondrial structural integrity in a Nrf2-dependent manner, likely due to its protective role against oxidative and inflammatory damage. Distinct from its role in anti-oxidative stress, Nrf2 has been shown to promote mitochondrial biogenesis. Pharmacological activation of Nrf2 could therefore upregulate both nuclear and mitochondria-encoded genes involved in mitochondrial function [3]. Regarding Nrf2 activation by mangiferin, we reasoned that mitochondrial biogenesis might be the potential mechanism by which it (mangiferin) improves mitochondrial integrity.

Under oxidative stress, mitochondrial integrity is impaired, and the loss of mitochondrial barrier allows for escape of mitochondrial content. Oxidative stress induces the formation of voltage-dependent anion channel (VDAC) oligomers in the mitochondrial outer membrane [22] and through this channel, mtDNA and proapoptosis proteins are effluxed [25]. Therefore, we reasoned that the mitochondrial protection conferred by mangiferin might be a key regulator in preventing mtDNA release.

Once released into the cytoplasm, mtDNA acts as a ligand of DAMP to elicit different inflammatory signaling cascades. TLR9 recognizes mtDNA as a ligand to activate NF-κB signaling [8]. Consistently, mtDNA, but not nDNA, has previously been reported to induce TLR9 activation and inflammatory response in mouse cardiomyocytes during hypoxia‐reoxygenation [26]. Also, cGAS binds to mtDNA and induces STING activation, resultantly inducing interferon response [7]. Mangiferin effectively blocked TLR9 and cGAS-STING signaling cascades, a process that is largely due to its ability to limit mtDNA release from mitochondria. The anti-inflammatory activities of mangiferin have been well documented. Our findings suggest that Nrf2 activation and mitochondrial protection are cardinal mechanisms that work in concert to mitigate inflammation in sepsis-induced heart injury. This conclusion is strongly supported by the fact that specific Nrf2 knockdown in the heart of septic mice diminished these beneficial effects of mangiferin.

## Conclusion

On the basis of the findings of this study, it can be concluded that through Nrf2 activation, mangiferin ameliorates mitochondrial dysfunction to block mtDNA release and subsequent cGAS-STING pathway-related inflammation. Taken together, these processes resultantly confer protection on the heart against sepsis-induced injury.

## Data Availability

The data produced from this study are available from the corresponding author on reasonable request.

## Abbreviations

cGAMP: Cyclic GMP-ATP
cGAS: Cyclic GAMP-AMP synthase
DAMPs: Damage-associated molecular patterns
IRF3: Interferon regulatory factor 3
Keap-1: Kelch-like ECH-associated protein 1
LDH: Lactate dehydrogenase activity
LPS: Lipopolysaccharide
mPTP: Mitochondrial permeability transition pore
mtDNA: Mitochondrial DNA
Nrf2: Nuclear factor-erythroid 2-related factor 2
OXPHOS: Oxidative phosphorylation
ROS: Reactive oxygen species
SDS-PAGE: Sodium dodecyl sulfate–polyacrylamide gel electrophoresis
STING: Stimulator of interferon genes
TBK1: TANK-binding kinase 1
VDAC: Voltage-dependent anion channel
